# Dynamic single-cell NAD(P)H measurement reveals oscillatory metabolism throughout the *E. coli* cell division cycle

**DOI:** 10.1038/s41598-018-20550-7

**Published:** 2018-02-01

**Authors:** Zheng Zhang, Andreas Milias-Argeitis, Matthias Heinemann

**Affiliations:** Molecular Systems Biology, Groningen Biomolecular Sciences and Biotechnology Institute, University of Groningen, Nijenborgh 4, 9747 AG Groningen, Netherlands

## Abstract

Recent work has shown that metabolism between individual bacterial cells in an otherwise isogenetic population can be different. To investigate such heterogeneity, experimental methods to zoom into the metabolism of individual cells are required. To this end, the autofluoresence of the redox cofactors NADH and NADPH offers great potential for single-cell dynamic NAD(P)H measurements. However, NAD(P)H excitation requires UV light, which can cause cell damage. In this work, we developed a method for time-lapse NAD(P)H imaging in single *E. coli* cells. Our method combines a setup with reduced background emission, UV-enhanced microscopy equipment and optimized exposure settings, overall generating acceptable NAD(P)H signals from single cells, with minimal negative effect on cell growth. Through different experiments, in which we perturb *E. coli*’s redox metabolism, we demonstrated that the acquired fluorescence signal indeed corresponds to NAD(P)H. Using this new method, for the first time, we report that intracellular NAD(P)H levels oscillate along the bacterial cell division cycle. The developed method for dynamic measurement of NAD(P)H in single bacterial cells will be an important tool to zoom into metabolism of individual cells.

## Introduction

Recent work has shown that individual microbial cells in a population can express different metabolic phenotypes^1–5^. For instance, different subpopulations of *E. coli* were observed in clonal populations growing in glucose^4^ or after a glucose-gluconeogenic carbon source shift^1^. While such phenotypic differences are typically identified with fluorescent proteins highlighting differences in protein expression, in most cases the true metabolic phenotype in these metabolically-divergent subpopulations – i.e. the metabolite levels – remains elusive. This is because methods to measure metabolites on the single cell level are largely lacking. Fluorescence resonance energy transfer (FRET) sensors are one of the very few methods for single-cell metabolite measurements^6–9^. However, these sensors often have a limited dynamic range and the fluorescence intensity of the fluorescent proteins can also be influenced by the physiochemical conditions in the cell (such as pH, oxygen level, ionic strength, etc)^10–13^. Therefore, FRET sensors may not be suitable for all studies; particularly those, where the physicochemical conditions of the cells would be perturbed are critical.

For quantifying the redox cofactors NAD(P)H in single cells, one can exploit the fact that their reduced forms fluoresce, when excited with light in the ultraviolet A range (UVA). While the use of these metabolites’ autofluorescence solves some of the problems associated with fluorescent proteins, the low quantum yield of the NAD(P)H fluorescence^14,15^ and the UVA-induced cell-damage^16^ represent other challenges, i.e. those connected with low intensity readouts, and potential cell growth defects in dynamic studies. Although some applications exist for dynamic single-cell NAD(P)H measurement in mammalian^17^ and yeast cells^18^, to the best of our knowledge, this method has not yet been used to study NAD(P)H levels in single live bacterial cells, where the challenges are even greater due to the very low amounts of NAD(P)H present in the small volumes of bacterial cells.

In this work, we developed a method to measure NAD(P)H levels dynamically in single live *E. coli* cells using the autofluorescence of NAD(P)H. Specifically, we developed a flow-channel for culturing *E. coli* with minimized background signal at the required wavelength, and exploiting UVA-optimized microscopy equipment (i.e. objective, camera and filters) we identified an excitation protocol able to generate acceptable fluorescence intensities, while limiting growth defects. Through metabolic perturbation experiments, we validated that the observed signal originates from NAD(P)H. Our method allows to determine NAD(P)H levels in single *E. coli* cells at a 10-min resolution for more than 20 hours with minimal effect on growth. Using this method, we found oscillations in the NAD(P)H levels in synchrony with the cell division cycle, suggesting fluctuating metabolic activity throughout the bacterial cell division cycle. We expect that our method for measuring NAD(P)H levels in single bacterial cells will be a valuable tool for investigations of metabolic heterogeneity.

## Results

### Optimal exposure settings to balance photo damage and signal intensity

The excitation wavelength of NAD(P)H ranges from 300 to 370 nm with a maximum at 340 nm^14^. Light of this wavelength falls into the ultraviolet A range (UVA), and is known to harm bacteria by damaging DNA or producing reactive oxygen species (ROS)^19,20^, resulting in decreased or halted growth^21–23^. Thus, towards dynamic NAD(P)H determination in live bacterial cells over multiple hours, we had to find ways to reduce the UVA exposure as much as possible while still generating sufficient signals. First, we optimized our imaging hardware. Specifically, we used excitation at 365 nm-light (FWHM: 8.46 nm), resembling a wavelength in the upper range of the excitation spectrum of NAD(P)H, and microscope hardware (i.e. objectives, filters and camera, see Materials and Methods), which all was optimized for increased transmission and sensitivity for the wavelengths for NAD(P)H excitation and emission.

Next, we needed a microfluidic setup that would generate as low as possible background intensity at the employed excitation and emission wavelengths. Here, comparing with conventionally used poly-acrylamide pads, we found that immobilizing *E. coli* on silanized cover glass, where a positively charged surface traps *E. coli* cells by electrostatic forces^24^, could generate 40% less background intensity at the respective wavelength (see Supplementary Fig. S1). By bonding a polydimethylsiloxane (PDMS) slab (with a single channel) onto the silanized cover glass, we fabricated a flow channel, through which perfusion of fresh growth medium would maintain constant growth conditions throughout the 20-hour observation period.

Using this imaging hardware and microfluidic setup, we then asked whether we could identify exposure settings including exposure time, interval and excitation power, allowing NAD(P)H measurements over multiple hours without or with only minimally harming cells. To identify such settings, we performed multiple experiments, in which we cultured *E. coli* on the abovementioned microfluidic device with continuous glucose minimal medium supply. In each experiment, we imaged with different exposure settings (exposure time * excitation power as measured at specimen, see Methods and Materials), and determined the resulting growth rates. The imaging was carried out with bright field and 365 nm-light once per 5, 10 or 15 min over a period of 20 hours.

**Figure 1:**
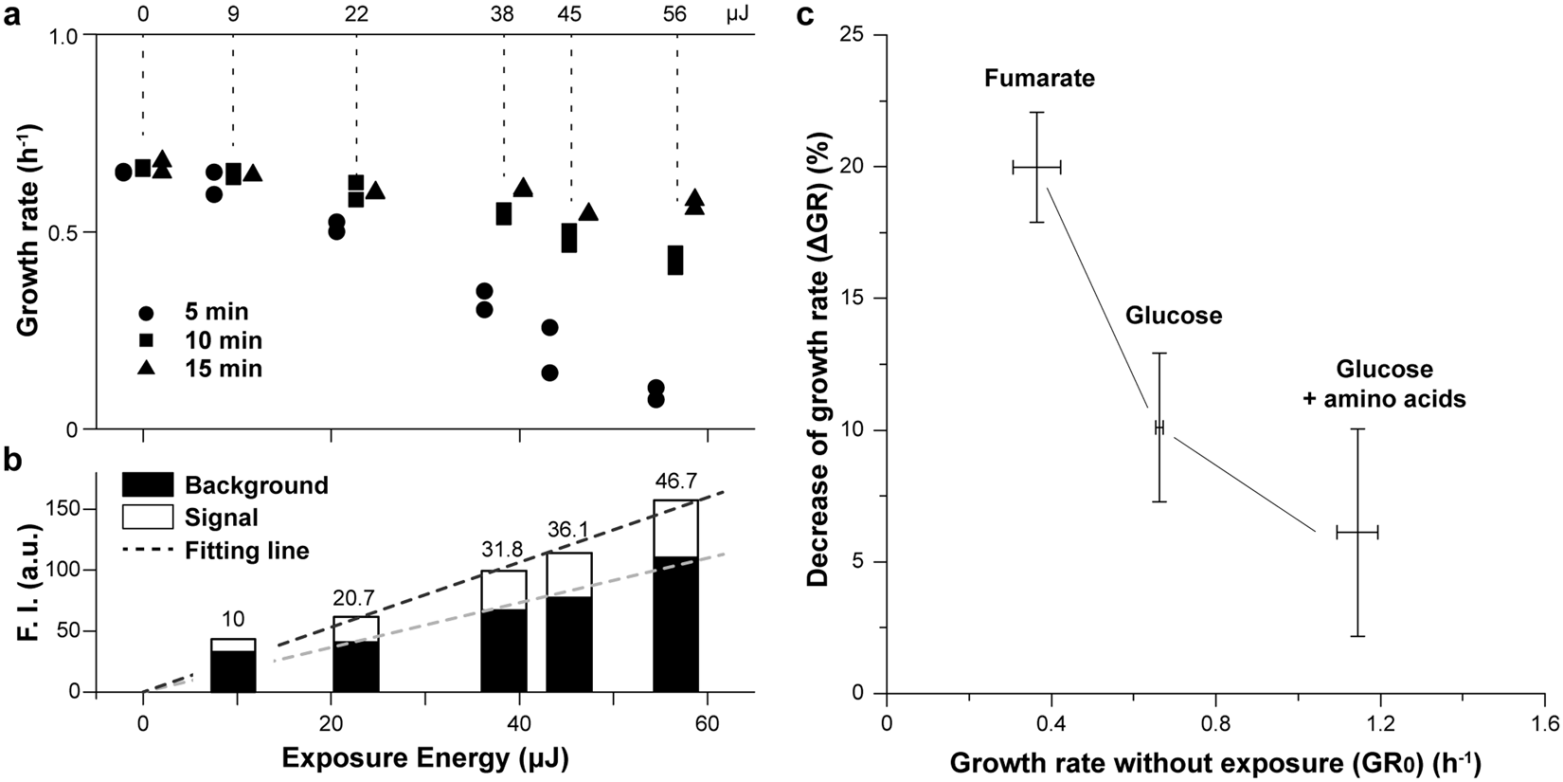
Influence of exposure energy and exposure interval on *E. coli* growth and NAD(P)H fluorescence intensity. (**a**) Growth rate of *E. coli* at different exposure energies and three exposure intervals. Between 4 and 10 hour after loading, *E. coli*’s growth rate at each exposure setting was determined from two replicates with at least 20 cell tracks in total, using exponential-fitting to cell sizes. Minimal medium with 5 g/L glucose was used as culture. For each replicate, the median growth rate is shown. Each symbol represents data from an independent experiment. Number of tracked cells in Fig. 1 can be found as Supplementary Table 1. (**b**) Fluorescence intensity from NAD(P)H autofluorescence and from background obtained with 5 different exposure energies and 10-min exposure interval. Every bar represents data from 2 independent experiments with at least 20 cell tracks in total. For each track between 4 to 10 hours, fluorescence intensities were obtained from inside of cells (denoted as signal) and surrounding area with no cells (denoted as background). The dynamics of the background intensity with time is shown in Supplementary Fig. S1b. In each replicate, signal and background intensities from single tracks were both averaged and the mean of both replicates are shown as bars for corresponding exposure settings. Values of signal intensity at every exposure settings are listed. At 9 μJ, the ratio of NAD(P)H intensity over background intensity was low compared to the higher exposure energies, suggesting that here the NAD(P)H signal was too low to be accurately distinguished from the camera noise. Linear fitting of background and signal intensity (excluding the 9 μJ group) are shown as dashed lines. (**c**) Decrease of growth rate by 365 nm-light exposure for *E. coli* growing in minimal medium with 2 g/L fumarate, 5 g/L glucose and 5 g/L glucose supplemented with 0.5% casamino acids. Cells were exposed with and without 365 nm-light in each growth medium in three replicates and the growth rates of at least 25 cell tracks were obtained for each medium in each replicate. 22 μJ/10 min was used as exposure program. In each replicate, the mean growth rate of un-exposed cells (*GR*_0_) was obtained and the decreases of exposed cells’ growth rate from *GR*_0_ were calculated and averaged, as *ΔGR*. The mean of *ΔGR* from all three replicates is shown with error bars indicating standard deviation, whereas the mean of *GR*_0_ in all three replicates is shown with error bars indicating one standard deviation.

Next, we segmented the acquired cell images by manually creating regions of interest (ROIs) based on the bright field images and tracked single cells throughout time, always following one of the two sister cells after each division. Note that with the used silanized cover glass as cell-attachment methods, cells are frequently lost after division. We found that the cells’ growth rates, as determined by the change of area (referred to as cell size hereafter) between 4 and 10 hours after the start of the experiment, were reduced at higher exposure energies (Fig. 1a). Up to an exposure energy of 9 μJ, all tested exposure intervals had the same influence on the growth rate but at higher exposure energies, imaging with 5-min interval further aggravated the growth rate reduction. Thus, growth defects occur at all tested exposure energies and intervals. However, the growth rate defect can be as low as 10% at 9 μJ and 15 min interval, but also as high as 89% at 56 μJ and 5 min. Notably, the observed decrease of growth rate was not due to the emergence of non-growing or dead cells, but to a reduction of growth rate across all cells (see Supplementary Fig. S1c).

Next, we asked how strong the signal from the intracellular NAD(P)H autofluorescence (denoted in the following as signal intensity) would be compared to the background signal. To assess this, we determined the fluorescence intensities of single cells and the background intensity. Specifically, the ROIs determined from the bright field images were applied to corresponding fluorescence images in the NAD(P)H-channel, while background fluorescence intensity (stemming from the cover glass, medium and PDMS, in the following denoted as background intensity) was determined from an area outside the ROIs. The mean intensity of each ROI was then used to indicate NAD(P)H autofluorescence intensity in single cells after subtracting the background intensity. Here, we found that above an exposure energy of 22 μJ, the intensity of the NAD(P)H autofluorescence was on average about 45% of the background intensity, as determined by linear regression (Fig. 1b), whereas at 9 μJ, this percentage was 33%. This is also consistent with the fact that at exposure energies of 22 μJ and higher we could observe the shapes of the cells from NAD(P)H autofluorescence. For this reason, we chose 22 μJ for the following metabolic perturbation experiments, although even higher exposure energies would better exploit the camera’s dynamic range, so that more details of fluorescence dynamics could be recorded. Thus, depending on the purpose of experiments, the applied exposure settings should represent a balance between compromises in terms of cell growth and signal intensity. The here presented results can serve as reference to determine such settings.

We next tested whether growth on different nutrients would lead to the same susceptibility to the 365 nm-light exposure as on the minimal glucose medium. Specifically, we applied the 22 μJ/10 min exposure program to *E. coli* growing in minimal medium with fumarate or with glucose supplemented with casamino acids. Here, we found that the growth rate reduction upon exposure anti-correlated with the growth rate without 365 nm-light exposure (Fig. 1c). This finding could be explained by the fact that within one division cycle in total more light is exposed to the slowly growing cells, thereby causing an increased growth rate reduction. Alternatively, it could be that the higher respiratory activity on fumarate compared to the other conditions is responsible for the increased growth defect on this carbon source. Importantly, these findings demonstrate that depending on the applied nutrient conditions, exposure settings would need to be adjusted to minimize growth defects.

Overall, we have shown that it is possible to obtained signals above the background, with using a background-minimized microfluidic setup, optimized imaging hardware and appropriate exposure settings. While growth defects cannot be fully avoided, they can be as low as 10% or lower. However, optimal exposure settings may need to be adjusted for different growth conditions.

### Metabolic perturbations indicate that the observed fluorescence stems from NAD(P)H

Although we used an emission filter with narrow passband (440–455 nm), which excluded most of the other UVA-excited autofluorescence sources of *E. coli* (such as flavins^25^), we still sought to validate that the observed fluorescence signal originated from NAD(P)H. Therefore, we metabolically perturbed cells, observed the response of single-cell signal intensities and benchmarked these responses with the expected NAD(P)H levels changes.

**Figure 2:**
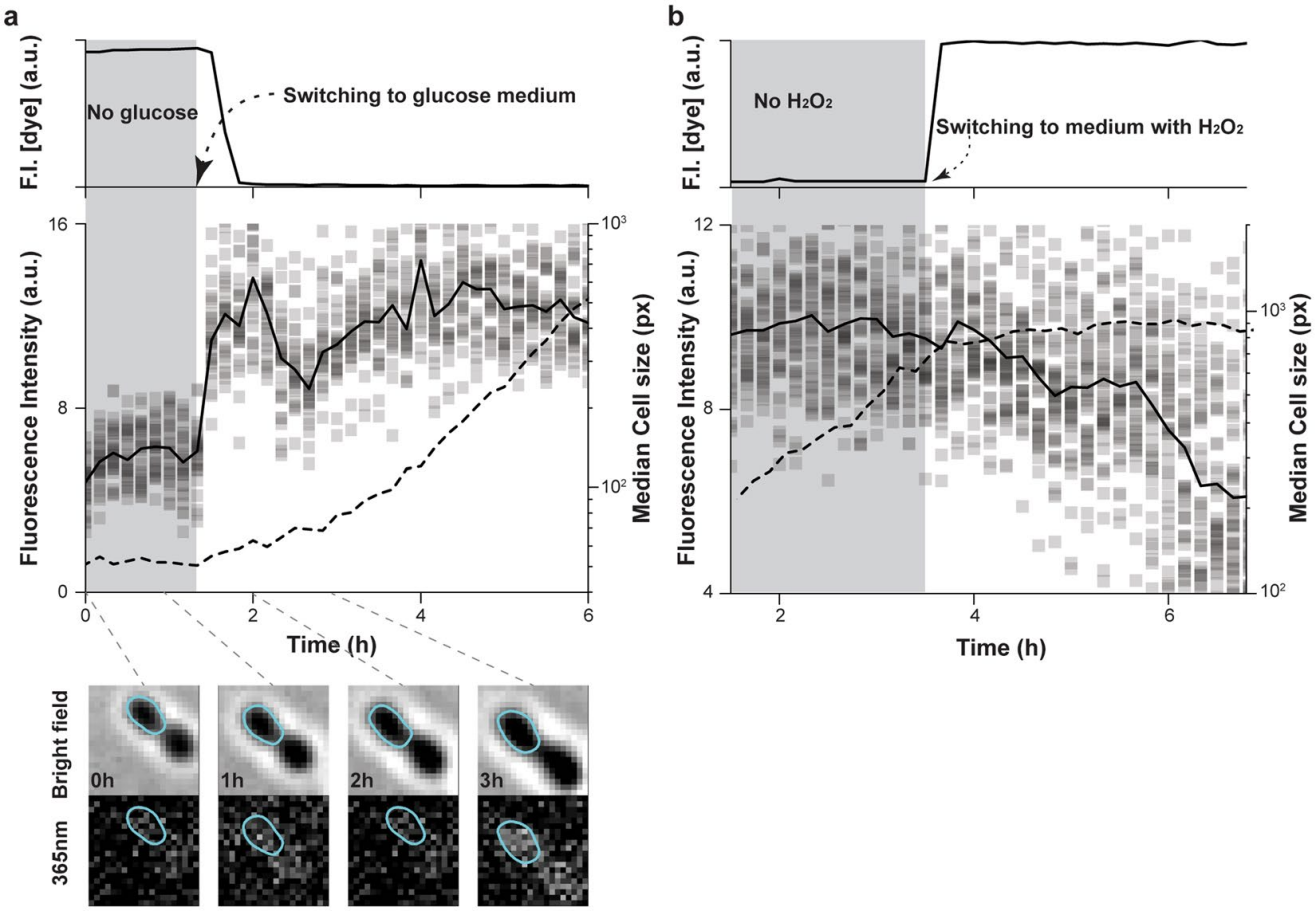
Changes of autofluorescence upon metabolic perturbations demonstrate that the observed fluorescence signals stem from NAD(P)H. (**a**) Supplying glucose to starved *E. coli* increased the fluorescence intensity. Cells were cultured in minimal medium without carbon source for 5 hours (gray shaded region) after having been loaded into the microfluidic device before glucose supply. The arrival of glucose-containing medium was indicated by the descent of fluorescence dye intensity. Note, due to experimental variances, between individual experiments it can take different amounts of time until the new medium reaches cells after a medium switch. 30 cells were tracked and their fluorescence intensity, the median of their fluorescence intensity and cell size are shown as squares, solid lines and dash lines, respectively. Four images illustrate the observed upshift of autofluorescence in NAD(P)H-channel, with corresponding images in the bright field. For a quantification of the fluorescence signal, see Supplementary Fig. S3. (**b**) Adding 0.4 mM H_2_O_2_ to glucose-grown *E. coli* reduced the fluorescence intensity. Cells were growing in minimal medium for 3.5 hours (gray shaded region) before 0.4 mM H_2_O_2_ was added into the flow-channel as indicated with arrow. The increase of the fluorescence dye intensity indicates arrival of the new medium at the cells. 48 cells were tracked and their fluorescence intensity, the median of their fluorescence intensity and cell size are shown as squares, solid lines and dash lines, respectively.

First, we added glucose to starved cells, where we expected to observe a fast increase in NAD(P)H levels as a result of the suddenly increased glycolytic flux^26^. Specifically, we cultured *E. coli* in our microfluidic chip for 5 hours in minimal medium without carbon source, but with a fluorescent dye. After switching to a second medium with glucose but without the dye, a drop of fluorescence from the dye indicated the arrival of new medium in the flow-channel. We found that the starved cells had a low fluorescence intensity in the NAD(P)H channel. After glucose supply, the fluorescence intensity increased 2-folds within 20 min and cells instantly started to increase in size (Fig. 2a). This change in fluorescence and the timescale of the increase reflects the expected response in the intracellular NAD(P)H levels upon this perturbation.

In a second perturbation experiment, we challenged *E. coli* with H_2_O_2_. As main cellular reducing force, NAD(P)H was expected to be utilized for neutralizing external H_2_O_2_, by for instance, the thioredoxin systems, glutathione system or AhpCF^27,28^, so that we anticipated a drop of the intracellular NAD(P)H levels upon H_2_O_2_-challenge. Specifically, after cells grew for 7 hours in glucose minimal medium, we supplemented the medium with a mixture of 0.4 mM H_2_O_2_ and a fluorescence dye to indicate arrival of H_2_O_2_ in the field of view. Here, in agreement with our expectation, we observed a drop of the NAD(P)H fluorescence intensity starting from 30 minutes upon H_2_O_2_ arrival (Fig. 2b). Consistently, by disrupting the function of AhpCF, no drop occurred (see Supplementary Fig. S2). Thus, since in these perturbation experiments the expected changes in fluorescence occurred within the expected timeframes, our findings strongly suggest that the fluorescence observed with our imaging method must stem from NAD(P)H.

### Oscillation in NAD(P)H levels during the cell division cycle

Using the capability of our method to determine intracellular NAD(P)H levels in single *E. coli* cells over long periods of time, we sought to investigate whether the NAD(P)H levels are constant throughout the cell division cycle, or whether they oscillate, as recently found in yeast^29^. For the analysis of the NAD(P)H dynamics along the bacterial cell division cycle, we cultivated *E. coli* in glucose minimal medium, acquired images every 10 minutes, and obtained single-cell trajectories of the NAD(P)H fluorescence levels between cell divisions.

Though the experiments were carried out in constant conditions (e.g. environmental temperature, excitation power, etc), the signals from single cells were noisy and sometimes exhibited long-term dynamics so that an obvious pattern of NAD(P)H level along the cell division cycle was hard to observe from individual cell trajectories (Supplementary Fig. S4a and b). Therefore, we next turned to data post-processing to reveal population-level trends. Specifically, a spline was fitted to the NAD(P)H signal trajectory of each cell (over a period of 10 hours), and a normalized trajectory was determined by dividing the original trajectory by this spline (Supplementary Fig. S4c and d). As division times vary between cells and cell division cycles, the trajectories over single division cycles contained different numbers of images. To enable averaging of the normalized trajectories, we generated 19 evenly spaced data points between both ends of each individual division cycle using linear interpolation. In this way, each interpolated single-cell trajectory comprised 21 data points (19 + 2 endpoints). Finally, by computing the median of these trajectories, we obtained an estimate of the average NAD(P)H levels during the cell division cycle.

**Figure 3:**
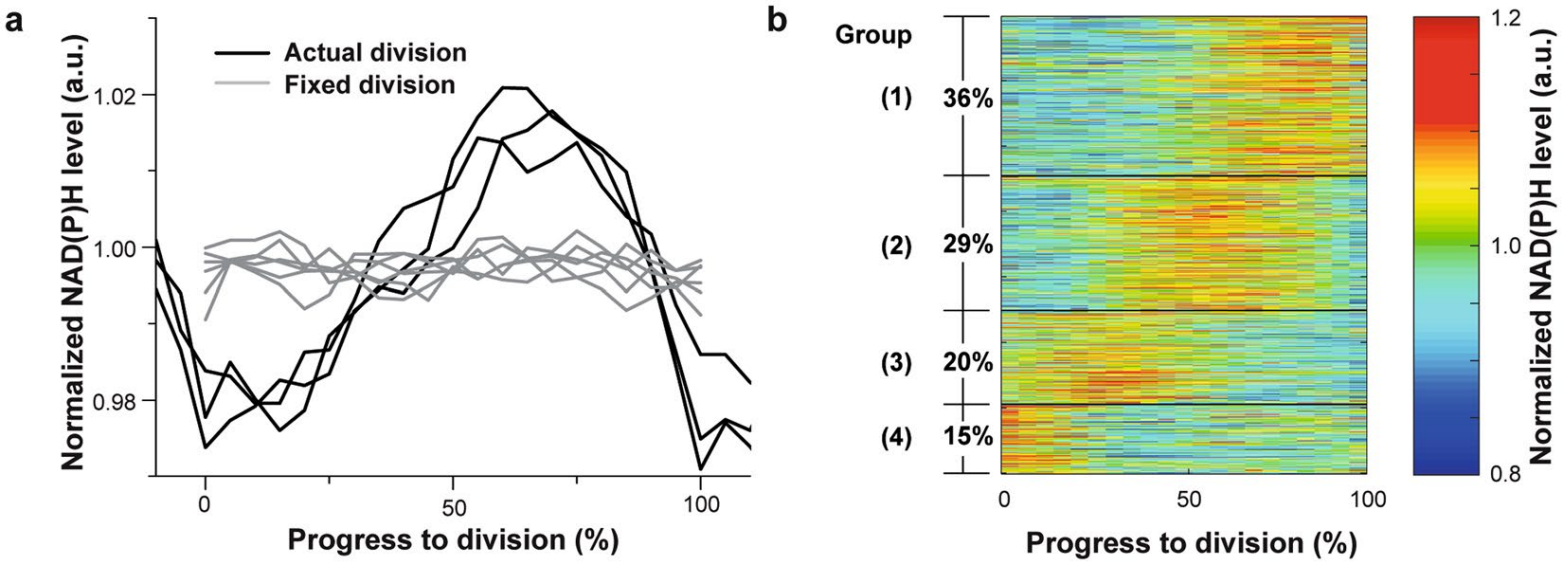
NAD(P)H levels show an oscillatory pattern throughout the *E*. *coli* cell division cycle. (**a**) Median of spline-normalized NAD(P)H levels along division cycles using actual and fixed division events. Trajectories were determined from 159 cells from three independent experiments, in which *E*. *coli* was grown in glucose minimal medium and imaged at 45 μJ every 10 minutes for 10 hours. 887 division cycles were identified and were used as actual division events while 60, 70, 80, 90, 100, 110 min were, respectively, used as fixed division times in the data processing (see Materials and Methods). Three median curves generated with actual division events in three experiments (including 10% of the adjacent division cycles) were plotted as solid lines, while median curves from the data of all three experiments were generated with fixed division events and showed as gray lines. Supplementary Fig. S4h shows an alternative normalization with a linear function fitted to the first and last intensity points of every division cycle (see Methods and Materials). (**b**) After processing with normalization and interpolation, trajectories of NAD(P)H levels in 887 division cycles from all three experiments were clustered with k-means (4 clusters, separated with thin black lines, performed with Matlab). The color scale indicates the NAD(P)H level relative to that at initial and ending points of division cycle. The upper two clusters (n = 575, 63% of all samples) have an increased NAD(P)H level in the middle of the cycle. Supplementary Fig. S4i shows the clustering results on the basis of the alternative normalization shown in Supplementary Fig. S4h.

Here, despite the noisy NAD(P)H level during individual division cycles, we found a clear ‘increase-drop’ oscillatory pattern in the average NAD(P)H levels across the cells division cycle (Fig. 3a). We next verified that the trend is repeated in adjacent division cycles, and is thus less likely to be a processing artifact. To do this, for each division cycle we investigated the NAD(P)H dynamics including the NAD(P)H data from the last part of the preceding and the initial part of the cycle that follows it (details provided in Materials and Methods). In this way, the descending trend in NAD(P)H levels from the last part of the previous cycle and the increasing trend in the initial part of the following cycle were clearly observable, providing further support to the observed NAD(P)H dynamics.

To still generate a visual impression of the single cell data, we resorted to a clustering analysis, using all normalized individual trajectories obtained in the three replicate experiments. This analysis revealed that in about 65% of the trajectories (Group 1 and 2 in Fig. 3b), the maximum NAD(P)H level was attained in the second half of their division cycles, which indicated that the maxima of NAD(P)H occur non-uniformly throughout the division cycles (Supplementary Fig. S4e). Similar percentages were also obtained when we analyzed the data from the three individual experiments separately. Additionally, the clustering analysis revealed that the fluctuation of NAD(P)H within division cycles in single cells was much more prominent than the median of the entire dataset as shown in Fig. 3a. Thus, despite the noise in the raw data, we were able to establish that the majority of cells displayed an ‘increase-drop’ oscillatory pattern of NAD(P)H during division cycles, which resulted in the observed average behavior.

Next, to verify the observed NAD(P)H pattern was not an artifact of the data analysis, we re-processed the raw NAD(P)H trajectories of single cells by assuming arbitrarily fixed division times. Specifically, as we found that 80% of the division cycles took 60–110 min, we selected six values from this range as fixed division times, segmented the single-cell NAD(P)H trajectories according to these division times, and processed these data with the same normalization and interpolation procedure as before. The obtained median curves were almost flat (gray lines in Fig. 3a), indicating that the originally obtained oscillatory pattern was not a result of our post-processing approach. This oscillatory pattern was also observed with another normalization method where a linear function was fitted to the first and last point of each single-cell trajectory (between two cell divisions, Supplementary Fig. S4c), and then the differences between the trajectory and this linear function were computed to yield a normalized trajectory for each cell (Supplementary Fig. S4h and i).

Since the division events were determined by morphology of cells in bright field images, we next verified if the accuracy of determining division events in this work could bias the pattern of NAD(P)H level along division cycle. Specifically, we applied a uniformly random plus/minus one frame or no frame shift at both ends to the individual division cycles, in order to check if the resulting median NAD(P)H levels in replicate experiments deviated from the observed ‘increase-drop’ pattern. Here, in all ten tests performed for each of three replicate experiments, the ‘increase-drop’ pattern remained (see Supplementary Fig. S4g), indicating that the determination accuracy of division events did not bias our results, therefore confirming that the oscillatory pattern in NAD(P)H levels must indeed occur during the *E. coli* cell division cycle.

## Discussion

In this work we established a method for dynamic single-cell NAD(P)H measurement in *E. coli* via the autofluorescence of NAD(P)H. In this method, we used a microfluidic flow-channel with low background intensity and a UVA-optimized microscopic setup, and developed exposure settings, which, on the one hand generate sufficient signal intensity from intracellular NAD(P)H autofluorescence and, result in limited cell growth defects on the other. By perturbing *E. coli*’s metabolism and redox state, we generated strong evidence that the acquired signal originates from NAD(P)H. Using this method, for the first time, we uncovered an oscillatory behavior of NAD(P)H levels throughout the *E. coli* cell division cycle.

While intracellular NAD(P)H measurement via autofluorescence was first proposed a long time ago^30^ and has been widely used in eukaryotes, to the best of our knowledge it has not yet been implemented for single prokaryotic cells. The most likely reason for this is that the small volume of prokaryotic cells which contains less amount of NAD(P)H than eukaryotes requires stronger excitation to generate visible signals, which in turn exerts severe negative effects on cell growth. For example, compared to our recent work in yeast^29^, a 4-fold higher excitation is necessary to image NAD(P)H in *E. coli* than in yeast. While this could be due to in general lower concentrations of NAD(P)H in *E. coli*, we rather feel that this has to do with the about 40-fold lower cell volume of *E. coli*. Nevertheless, by carefully quantifying the impairment of *E. coli* cell growth and the intracellular NAD(P)H autofluorescence under various exposure energy and intervals, we demonstrated that, with appropriate exposure settings, dynamics of intracellular NAD(P)H in single bacterial cells can indeed be acquired using our method with limited cell damage from the excitation light. Notably, if the both redox cofactors would need to be measured separately, then fluorescence lifetime imaging microscopy (FLIM)^17^ would need to be exploited.

Recent work has revealed that the abundances of many biomolecules are oscillating with bacterial division cycle, including metabolites (e.g., cyclic-di-GMP as measured with liquid chromatography-tandem mass spectrometry on cell extracts of synchronized cultures^31^ or time-lapse microscopy with a fluorescence sensor^32^) and NAD(H)-dependent proteins like KidO and GdhZ^33,34^, suggesting a dynamic metabolism also in bacteria. Oscillating NAD(P)H levels along cell cycle, though reported in yeast^29^, has, to the best of our knowledge, never been observed in bacteria.

The observed ‘increase-drop’ oscillatory pattern of NAD(P)H during the *E. coli* cell division cycle could be caused by metabolic reactions in the second half of the division cycle, that consume NAD(P)H faster than it is regenerated by metabolism. Pioneering work showed that the production rate of phospholipids increases during the elongation phase (D-period) of bacterial division cycle^35,36^, which requires synthesis of fatty acids. In *Bacillus subtilis*, synthesis of phospholipid and fatty acids were even found to be coordinated with Z-ring formation^37^. Given the fact that biosynthesis of fatty acids in bacteria (type II fatty acid synthesis) consumes both NADH and NADPH in elongating the acyl chains^38,39^, one could speculate that the decrease in the second half of the division cycle (as visible in the overall trend, Fig. 3a) could be caused by increased fatty acid biosynthetic activity. However, since the intracellular NAD(P)H levels may also be affected by fluctuations in the activity of various metabolic pathways, the resulting NAD(P)H pattern in single cell cycles may be different.

Overall, our work provided a method for the investigation of intracellular NAD(P)H in bacteria, with which the dynamics of NAD(P)H level between *E. coli*’s division cycles was studied. The discovery of the oscillatory pattern in NAD(P)H during the *E. coli* division cycle is a good starting point for further investigation how metabolism operates throughout the cell division cycle in prokaryotes, eventually together with dynamic observations of other aspects, such as membrane voltage^40,41^. Finally, we envision that the developed method for dynamic measurement of NAD(P)H in single bacterial cells will in general be an important tool for zooming into metabolism of single bacterial cells.

## Materials and Methods

### Bacterial strain and growth conditions

BW25113 wild type *E. coli* was used in this work. M9 minimal medium^1^ with 5 g/L glucose was used in batch cultures and microscopic experiments, unless indicated otherwise. For testing growth with amino acids, the aforementioned medium were supplemented with 0.5% casamino acids (BD Bioscience). In experiments using fumarate as carbon source, glucose was replaced by 2 g/L fumarate. Stock solution of glucose and fumarate were prepared with MilliQ, filtered through 0.2 μm PES filter and the pH was adjusted to 7 with NaOH or HCl.

All bacterial cultures used in time-lapse movies were from single colonies and were firstly grown overnight at 37 °C, shaking at 300 rpm in a 500 mL flask with 50 mL medium. Sponge caps were used with flasks to enable sufficient oxygen supply in culture. In experiments with glucose as carbon source, the culture was diluted when optical density (OD_600_) was greater than 0.5 and then was further diluted 2 times in the course of the following 24 hours whenever OD_600_ reached 0.5. The growth rate in shaking flasks were between 0.6 and 0.66 hour^−1^ with glucose as carbon source while became 1.2 hour^−1^ if casamno acids were added. Once the final culture reached OD_600_ 0.5, cells were diluted 10 times (OD_600_ 0.05) in pre-warmed medium and loaded onto the microfluidic device. Here, the growth rate in flasks was between 0.4 to 0.44 hour^−1^ with fumarate as carbon source. In experiments using fumarate as carbon source, after a first overnight culture, the culture was diluted in M9 with 2 g/L fumarate, cultured until OD_600_ reached 0.2 and further diluted 2 times in the same way as done in experiments with glucose. When the final culture reached OD_600_ 0.2, the culture was diluted 4 times in M9 with fumarate for loading onto the microfluidic device. In the glucose perturbation experiments, *E. coli* were cultured in M9 with glucose as described before. After the final culture reached OD_600_ 0.5, 30 mL culture was harvested and centrifuged (5 min, 4000 g) at room temperature. Then, the pellets were re-suspended in 50 mL M9 without carbon source (500 mL flask) and incubated (300 rpm, 37 °C) for 4 hours, before being diluted to OD_600_ 0.05 for microscopy.

### Fabrication of microfluidic device

The microfluidic device used in this work is a polydimethylsiloxane (PDMS) slab bonded to a silanized cover glass. PDMS (Sylgard 184; Dow Corning) was poured onto a silicon wafer molded with a channel (dimensions: 50 μm (Width) × 5 μm (Height) × 1 cm (Length)) and then baked for 1 hour at 120 °C and 1 hour at 100 °C.

Silanization of cover glasses were performed at room temperature. Cover glasses (22 × 40 mm, Thermo Scientific) were sonicated in 5 M KOH for 30 min in a polytetrafluoroethylene container before being washed 10 times with MilliQ water. Then, the cover glasses were incubated with 2% (v/v) (3-Aminopropyl)triethoxysilane (APTES; Sigma) for 2 hours. Finally, the cover glasses were sequentially washed with MilliQ for 10 times, aceton for 2 times and were dried by compressed air. Processed cover glasses were kept in vacuum as moisture in the air deteriorates the APTES coating. Thus, microfluidic devices were fabricated right before experiments. Specifically, PDMS slab and silanized cover glass were irradiated with UV for 10 minutes before bonding together and baking at 120 °C for 1 hour.

### Microscopic setup

All microscopy was performed with a Nikon Ti-E inverted microscope, operated using NIS-Elements. Light sources for bright field and fluorescence imaging were a halogen lamp and an LED (pE-2, CoolLED), respectively. 365 nm-light was for excitation in NAD(P)H-channel with a filter set comprising a 350/50-nm band-pass filter, a 409-nm beam-splitter and a 435/40-nm emission filter while 470 nm-light was for the fluorescence dye with a filter set comprising a 470/40-nm band-pass filter, a 495-nm beam-splitter and a 525/50-nm emission filter. A wavelength-optimized 100x objective (MRF02900, Nikon) and camera (DU-897 EX, Andor) were used for image acquisition. The EM Gain Multiplier of the camera was disabled and the Readout Mode was set to 1 MHz to reduce the camera readout noise. The baseline level of camera was set to 500 at −75 °C to avoid negative intensity value. Exposure energies were determined as exposure time (set in NIS elements) multiplied by exposure power, which was measured with a power meter (PM100USB, Thorlabs) and a power sensor (S120VC, Thorlabs), located at the specimen plane. The Nikon Perfect Focus System was used in all time-lapse movies to ensure focusing. Images in bright field channel were focused 1.05 μm higher than that in the fluorescence channels to facilitate cell segmentation.

### Microfluidic experiments

All microscopic experiments were performed at 37 °C. In all time-lapse movies, fresh minimal medium was perfused into the chip at 6 μL/min (syringe pump in some experiments, air-pressurized pumping system in others) for 10 min to flush the flow-channel, followed by manually injecting diluted *E. coli* culture (OD_600_ 0.05) via a 1 mL syringe. Then, the medium flow rate was reduced to 0.5 μL/min for 3 min so that cells had a higher chance to attach to the glass surface. Finally, the flow rate was set to 3.4 μL/min to flush away loosely attached cells, to prevent later formation of colonies with multiple layer of cells. In experiments optimizing the exposure settings, perfusion was performed with a syringe pump, while in the experiments to probe metabolic perturbation and to test for NAD(P)H oscillations, an air-pressurized pumping system (OB1, Elveflow) was used together with a flow sensor (MFS2, Elveflow) for precise flow-rate control.

In the experiments optimizing the exposure settings, time-lapse movies in bright field and NAD(P)H-channel started 1 hour after loading cells. In the metabolic perturbation experiments, we used bright field and NAD(P)H-channel (22 μJ per 10 min), together with 470 nm (9 μJ per 10 min) to excite a fluorescent dye, 5-(and-6)-Carboxy-2′,7′-Dichlorofluorescein (C368; Thermo Fisher) mixed in medium. Medium switches were done manually by cutting and reconnecting tubings. In the glucose perturbation experiments, after loading starved cells onto the microfluidic chip, we continued to feed them with medium without carbon source (but mixed with the fluorescent dye) for additional 3 hours. Image acquisition started 1 hours after loading, meaning that cells were imaged for 2 hours before the medium switch. In the H_2_O_2_-perturbation experiments, cells were first cultured on chip in glucose minimal medium for 3.5 hours without any light exposure before image acquisition started. Medium containing H_2_O_2_ and the saturated water solution of abovementioned fluorescent dye was connected to the chip 3.5 hours after image acquisition started. To avoid deterioration of H_2_O_2_, two times concentrated H_2_O_2_/water solution and minimal medium were loaded separately in two 15 mL falcon tubes and were both driven into chip at the same flow rate. The two media converged in a connector before running into chip. Due to its instability, H_2_O_2_ solution was freshly prepared before experiment and the concentration was titrated with 2 mM KMnO_4_.

In experiments observing NAD(P)H along cell division cycles, bright field and NAD(P)H-channels were included in the time-lapse acquisition. 45 μJ (per 10 min) was used as exposure energy for NAD(P)H-channel to increase the resolution of NAD(P)H dynamics.

### Determining growth rate and intensity of NAD(P)H autofluorescence

In the experiments optimizing exposure settings and perturbing metabolism, cells were segmented manually into region of interests (ROIs) according to the bright field images. In the experiments to test for NAD(P)H oscillations we used MicrobeJ^42^, a plug-in for ImageJ, to segment cells from bright field images. To determine growth rates, the ROI areas were used. When a cell divided, the ROI area was doubled. Growth rates were calculated by fitting an exponential function to the time course (from hour 4 to 10) of the ROI areas.

To obtain the NAD(P)H fluorescence intensity, the ROIs were transferred to the corresponding images in the NAD(P)H-channel. In experiments optimizing exposure settings and perturbing metabolism with glucose, NAD(P)H fluorescence intensity were determined by subtracting mean fluorescence intensity of surrounding area with no cells from mean fluorescence intensity in ROIs. In all other experiments, NAD(P)H fluorescence in ROIs were obtained from fluorescence images which were corrected for background and uneven illumination. To do this correction, we included one to three ‘blank positions’ in time-lapse acquisition where no *E. coli* or fluorescent debris were present. Then, for every acquisition round, images from the blank positions were averaged and used for correcting images from experimental positions (with *E. coli*) as follows,

1 $${\rm{Corrected}}\,{\rm{image}}\,=\frac{{\rm{experimental}}\,{\rm{image}}\,-\,{\rm{blank}}\,{\rm{image}}}{{\rm{blankimage}}\,-\,{\rm{CCD}}\,{\rm{baseline}}\,{\rm{level}}}\times {\rm{mean}}\,{\rm{intensity}}\,{\rm{of}}\,{\rm{blank}}\,{\rm{image}}$$

where the CCD baseline level is an electronic offset added to the output signal of CCD sensor and was set to 500 in our work.

### Data processing for investigating NAD(P)H level in cell division cycles

In the experiments investigating the NAD(P)H levels along the cell division cycle, the mean fluorescence intensity in ROIs were used to quantify intracellular NAD(P)H level. To obtain this value, ROIs determined from bright field images were transferred to the background-corrected fluorescence images and pixels in every ROI whose intensity was lower than background noise value (10 in this work) were removed. Specifically, to determine this background noise value, we first selected an acquisition position and determined an area (bigger than 5000 pixels) containing no cells on all time-lapse images of this position. Then, all pixel values in this area on background-corrected fluorescence images were sorted and an intensity value that is greater than 90% of the selected pixels was determined as background noise for each frame and the median of these values was set as background noise for the experiment. Then, a normal probability distribution function was fitted from the rest of pixel intensities, by which the mean value of the function was obtained accordingly. Data in the first 10 hours were used in calculation.

To obtain trajectories of intracellular NAD(P)H levels between cell division events, single cells (or one of two sister cells after division) were tracked for 10 hours and the NAD(P)H signals from each cell track were then separated according to division events, as shown in Supplementary Fig. S4a,b and f. To remove the effect from long-term dynamics of NAD(P)H to NAD(P)H trajectories between cell division, we fitted a spline (smoothing parameter: 0.004) to every NAD(P)H trajectory of single tracks and the normalized trajectory is the quotient of original trajectory divided by the spline (see Supplementary Fig. S4c and d). NAD(P)H trajectories of cell division cycles was obtained according to cell division. As an alternative method for normalization, we normalized every original NAD(P)H trajectory for intensity by fitting a linear function to the first and last intensity points of every division cycle (dash line in Supplementary Fig. S4f), subtracting this function from original trajectory and using the residue as normalized trajectory of NAD(P)H level. With both normalization methods, to obtain the median trajectories, 19 time points were linearly interpolated between first and last time point of every normalized trajectory so that every NAD(P)H trajectory contained 21 time points in one division cycle.

To check if the observed pattern of NAD(P)H level in the current division cycle also appears in adjacent cycle, we first selected the NAD(P)H trajectories from the last time point of previous cycle to the first time point in the next cycle and normalized it based on the intensity profile in current cycle. Then interpolation was performed on the current cycle and the same interpolation interval were applied to the parts of trajectory outside of the current cycles. Finally, the mean of these ‘elongated’ NAD(P)H trajectories were calculated and shown in Fig. 3a.

### Calibration of intracellular NAD(P)H concentration in *E. coli*

To calibrate the intracellular NAD(P)H concentration in *E. coli* on our setup, we used NADPH-water solutions (note the similar spectral properties of NADH and NADPH). Specifically, 0.2, 0.3, 0.5, 0.75, 1.0 mM NADPH standard solutions were prepared by dissolving NADPH tetrasodium (Oriental Yeast) into MilliQ water and the concentration was verified spectrophotometrically at 340 nm using the known extinction coefficient of NAPDH. Wild-type *E. coli* was cultured in shaking flask as described. When the OD_600_ reached 0.5, growth medium was diluted 50 times with minimal medium without carbon source. The cultures was kept for further 6 hours while shaking. Cells were then loaded into microfluidic device and immobilized in the flow channel. MilliQ water was perfused into the chip while images were acquired in the brightfield and NAD(P)H-channels. This step was repeated using aforementioned NADPH standard solutions, one at a time. When the intracellular fluorescence intensity equaled the intensity of the surrounding area with no cells, the intracellular NAD(P)H concentration was considered identical to the one of the NADPH standard solution.

To determine fluorescence intensities, we manually segmented cells into region of interests (ROIs) according to the bright field images, which were then transferred to the corresponding images in NAD(P)H-channel. The difference of fluorescence intensity between cells and nearby areas was obtained for each analyzed cell by subtracting mean fluorescence intensity of surrounding area with no cells from mean fluorescence intensity in its ROI.

### Data availability statement

The datasets generated during and/or analyzed during the current study are available from the corresponding author on reasonable request.

## Electronic supplementary material

Supplementary Information
