# Supplementary material for: Dynamic single-cell NAD(P)H measurement reveals oscillatory metabolism throughout the E. coli cell division cycle
Source: Sci Rep. 2018 Feb 1;8:2162. doi: 10.1038/s41598-018-20550-7 (PMC5795003; doi:10.1038/s41598-018-20550-7)
Supplement: Supplementary file 1 — Supplementary Information [file 41598_2018_20550_MOESM1_ESM.docx]

**Supplementary Information for**

**Dynamic single-cell NAD(P)H measurement reveals oscillatory metabolism throughout the *E. coli* cell division cycle**

Zheng Zhang, Andreas Milias-Argeitis, Matthias Heinemann

1. Supplementary Figure S1

2. Supplementary Figure S2

3. Supplementary Figure S3

4. Supplementary Figure S4

5. Supplementary Table S1

**Supplementary Figure S1.**


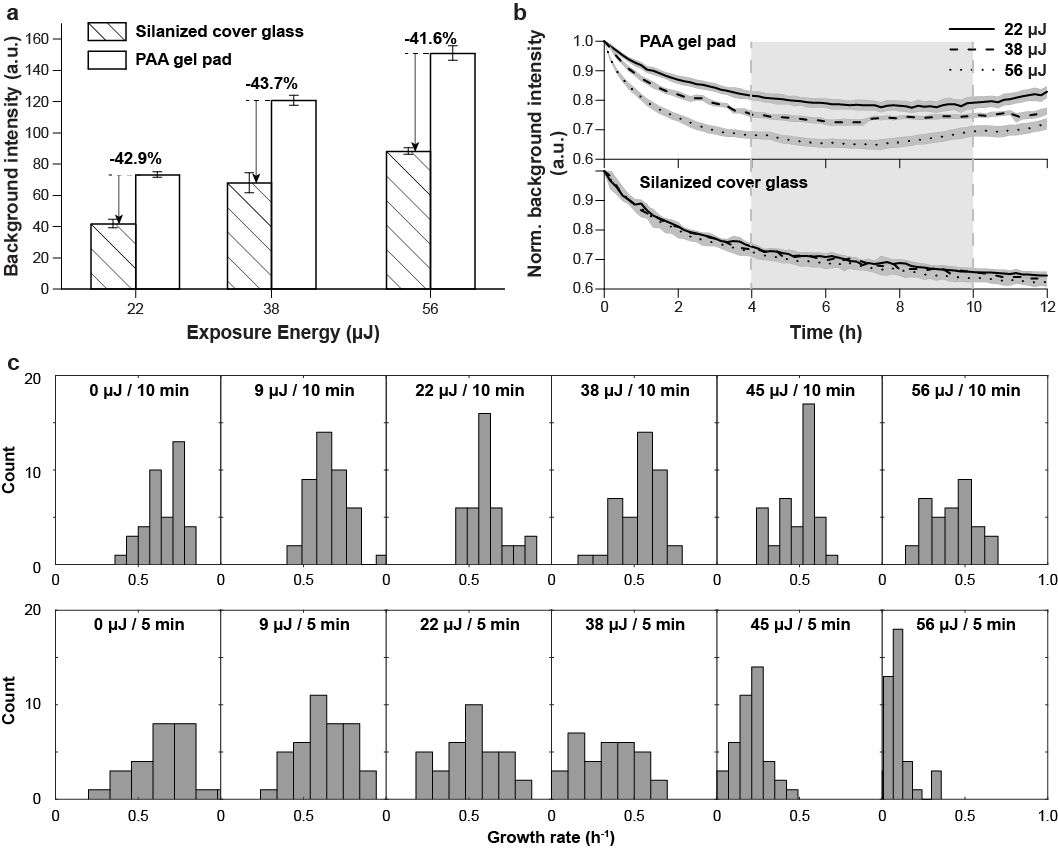


**Supplementary Figure S1. Background intensity from two setups and the reduction of grow rate from 365 nm-light exposure.**

(a) Comparison of background intensity generated from silanized cover glass setup and poly-acrylamide (PAA) gel pad setup with three exposure energies. Time-lapse movies were performed in both setups with indicated exposure energies and 10-min acquisition interval. At least 7 acquisition positions were selected and background intensity on images between 4 and 10 hour (gray shadow in Fig. S1b) were averaged for each position. Bar plots are the median of these averaged intensities with standard deviation as error bars. Percentages indicate the drop of intensity from that in PAA gel pad setup.

(b) Reduction of background intensity from 365 nm-light exposure over time in two setups with 3 exposure energies. The first intensity value for every acquisition position was divided by the rest to normalize. Median value at each time point was plotted with standard deviation shown as shadows along with median curves. Images between 4 and 10 hour (shown as gray shadow) were used in calculation for Fig. S1a.

(c) 365 nm-light-caused decrease of growth rate. Growth rates in experiments with 10-min and 5-min interval and specified exposure energies (shown as energy / interval) are plotted as histogram (n>40, from 2 replicates for each group). Unimodal distribution in each plot indicates that the 365 nm-light exposure equally decreases growth rates of all observed cells.

**Supplementary Figure S2.**


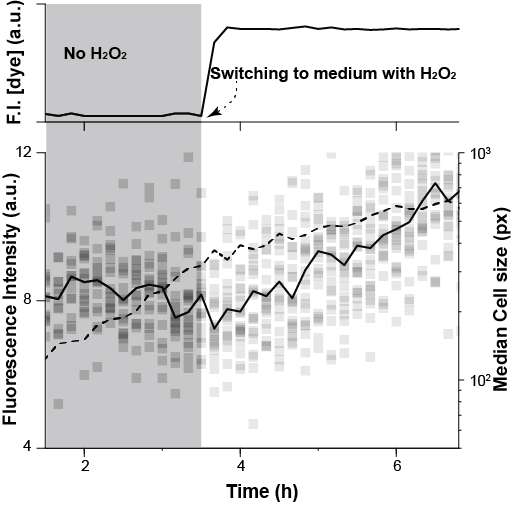


**Supplementary Figure S2. NAD(P)H level increased upon H_2_O_2_-stress in Δ*ahpC* strain.**

Adding 0.4 mM H_2_O_2_ to glucose-grown Δ*ahpC* cells elevated the fluorescence intensity. Cells were grown in minimal medium for 3.5 hours (gray shaded region) before 0.4 mM H_2_O_2_ was added into the flow-channel as indicated with arrow. The increase of the fluorescence dye intensity indicates arrival of the new medium at the cells. 25 cells were tracked and their fluorescence intensity, the median of their fluorescence intensity and cell size are shown as squares, solid lines and dash lines, respectively.

**Supplementary Figure S3.**


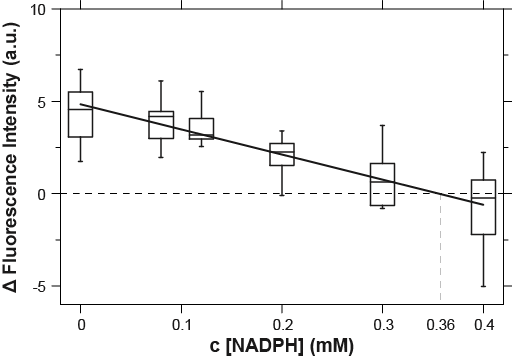


**Supplementary Figure S3. Calibration of intracellular NAD(P)H concentration in *E. coli***

Cells in minimal medium without carbon source were loaded into the microfluidic device and immobilized. Water and NADPH-water solutions (with 0.08, 0.12, 0.2, 0.3, 0.4 mM NADPH) were sequentially perfused into the chip while images of cells were taken in the bright field and NAD(P)H-channel. With each medium composition, at least 12 cells were identified and the difference of fluorescence intensity between cell and nearby area determined and plotted as box plot with the whiskers indicating the 10-90% percentiles. A linear line was fitted to the median values (r^2^=0.98) and the intercept on x-axis was indicated.

**Supplementary Figure S4.**


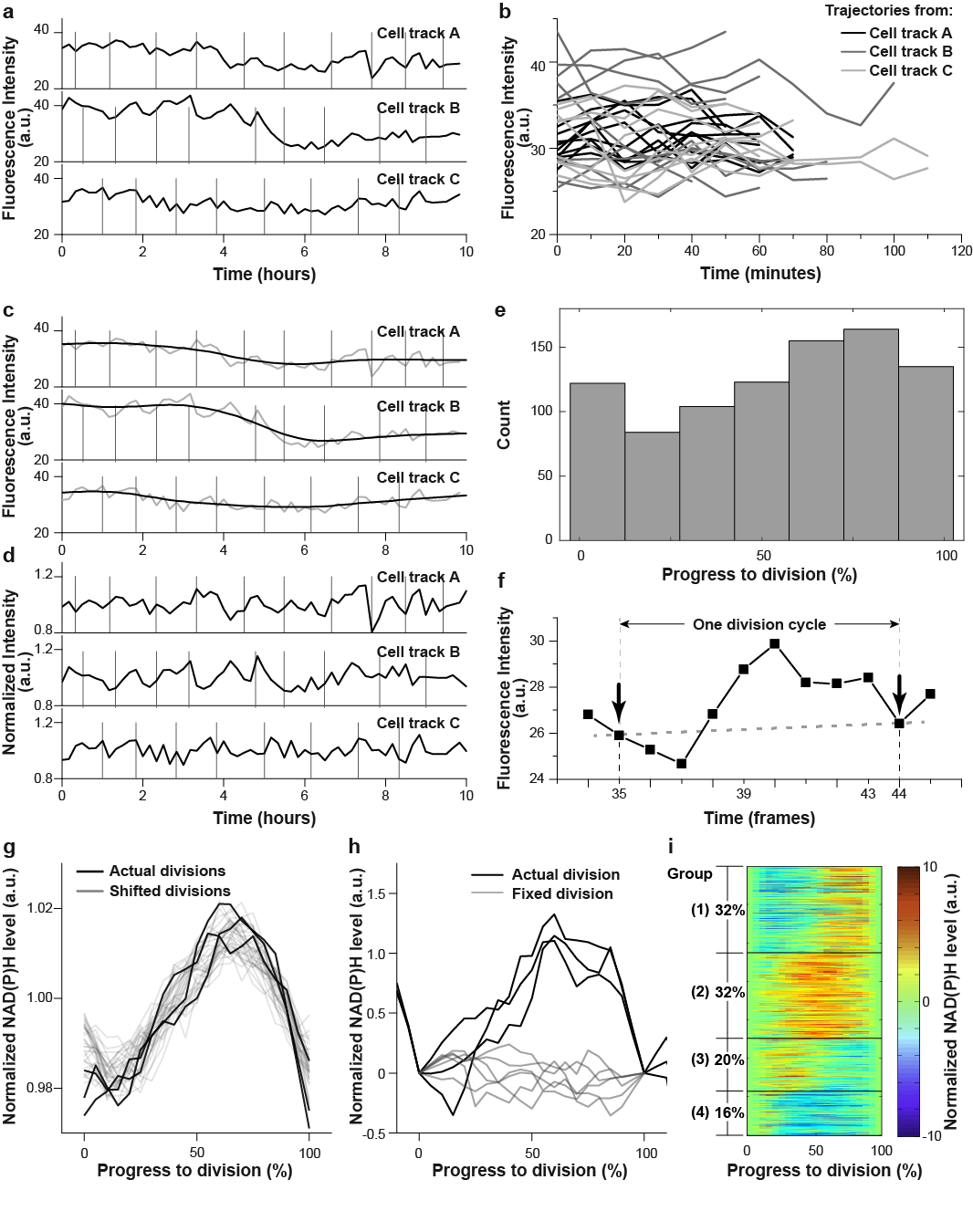


**Supplementary Figure S4. Dynamics of intracellular NAD(P)H level.**

(a) NAD(P)H levels in three single cell tracks. Upon cell division (vertical lines), one of two descendant cells were tracked.

(b) Data from (a) where here each line represents data from one division cycle. Time point 0 represents the moment of a cell division.

(c) Spline fitted to cell tracks in (a). (smoothing parameter: 0.004, performed by fitting smoothing spline in Matlab.)

(d) Normalized trajectories of cell tracks in (a). Normalized trajectories were obtained from dividing original intensity trajectories (a) by fitted spline (c).

(e) Uneven distribution of the times of maximum NAD(P)H level throughout the division cycle. For every trajectory in Figure 3, the time (normalized to the division cycle duration) when the highest NAD(P)H level occurred is located and used to generate the histogram.

(f) Fluorescence intensities from ROIs in one division cycle and one additional frame from each of the two adjacent cycles. The frames where cell division happened are marked with arrows. 45 μJ per 10 minute was used as exposure settings.

(g) With spline-fitting, median of normalized NAD(P)H levels along division cycles using actual and shifted division events. Each solid lines is from one replicate experiment, as also shown in Fig. 3a. For trajectories in each replicate, starting and ending divisions were randomly shifted one frame earlier, or not shifted or one frame later to test if the observed trend was altered. 10 such tests were performed for each of three replicate experiments and the resulting median curve of NAD(P)H level were plotted as gray lines (n=30).

(h) Median of normalized NAD(P)H level using actual and fixed division events. The same data in Fig. 3a except the normalization method was line-fitting, as described in Materials and Methods.

(i) The same data in Fig. 3b except the normalization method was line-fitting, as described in Materials and Methods.

**Supplementary Table S1. Number of cells tracked in Fig. 1.** For each combination of UV-dose and exposure interval in Fig. 1a, two replicates were performed and the numbers of tracked cells in each replicates were listed, separated by a comma.

| **Figure 1a and 1b** | | | | | | | **Figure 1c** | | | | | | |
| --- | --- | --- | --- | --- | --- | --- | --- | --- | --- | --- | --- | --- | --- |
| **UV-dose (J)** | **0** | **9** | **22** | **38** | **45** | **56** | **Carbon source** | **Glucose** | | **Fumarate** | | **Glucose + amino acids** | |
| Exposure interval  **5 min** | 9, 17 | 24, 18 | 14, 22 | 12, 21 | 17, 24 | 22, 20 | **UV-exposure** | **No** | **Yes** | **No** | **Yes** | **No** | **Yes** |
|  |  |  |  |  |  |  | **Replicate 1** | 20 | 7 | 15 | 15 | 17 | 13 |
| **10 min** | 20, 20 | 34, 8 | 7, 34 | 20, 20 | 5, 37 | 16, 20 | **Replicate 2** | 20 | 34 | 16 | 16 | 13 | 16 |
| **15 min** | 23, 21 | 5, 35 | 24, 16 | 8, 31 | 17, 23 | 6, 20 | **Replicate 3** | 20 | 20 | 35 | 53 | 17 | 30 |
| **Figure 1b** | - | 42 | 41 | 40 | 42 | 36 |  |  |  |  |  |  |  |

For each combination of UV-dose and exposure interval in Fig. 1a, two replicates were performed and the numbers of tracked cells in each replicates were listed, separated by a comma.
